# Analysis of the potential of human cultured nasal epithelial cell sheets to differentiate into airway epithelium

**DOI:** 10.1096/fba.2022-00106

**Published:** 2022-12-19

**Authors:** Yoshiyuki Kasai, Tsunetaro Morino, Tsuguhisa Nakayama, Kazuhisa Yamamoto, Hiromi Kojima

**Affiliations:** ^1^ Department of Otorhinolaryngology The Jikei University School of Medicine Tokyo Japan; ^2^ Department of Otorhinolaryngology, Head and Neck Surgery Dokkyo Medical University Tochigi Japan

**Keywords:** air‐liquid interface culture, cultured nasal epithelial cell sheet, differentiation potential, mucociliary function, multiciliated cell

## Abstract

Understanding the expected efficacy and safety of a new regenerative therapy requires analysis of the fate of the transplanted cell graft. We have shown that transplantation of autologous cultured nasal epithelial cell sheets onto the middle ear mucosa can improve middle ear aeration and hearing. However, it remains unknown whether cultured nasal epithelial cell sheets have the potential to gain mucociliary function in the environment of the middle ear because sampling cell sheets after transplantation is challenging. The present study re‐cultured cultured nasal epithelial cell sheets in different culture media and evaluated whether the sheets have the potential to differentiate into airway epithelium. Before re‐cultivation, cultured nasal epithelial cell sheets fabricated in keratinocyte culture medium (KCM) contained no FOXJ1‐positive and acetyl‐α‐tubulin‐positive multiciliated cells or MUC5AC‐positive mucus cells. Interestingly, multiciliated cells and mucus cells were observed when the cultured nasal epithelial cell sheets were re‐cultured in conditions that promote differentiation of airway epithelium. However, multiciliated cells, mucus cells and CK1‐positive keratinized cells were not observed when cultured nasal epithelial cell sheets were re‐cultured in conditions that promote epithelial keratinization. These findings support the suggestion that cultured nasal epithelial cell sheets have the ability to differentiate and gain mucociliary function in response to an appropriate environment (possibly including the environment found in the middle ear) but are unable to develop into an epithelial type that differs from its origins.

## Graphical abstract

Nasal mucosal epithelial tissue originally has multiciliated cells and mucus cells whereas there were no multiciliated cells, mucus cells in cultured nasal epithelial cell sheet. While cultured nasal epithelial cell sheet has the ability to differentiate into airway cells and stratified squamous cells along with surrounding condition, it could not differentiate into keratinized cells probably because of memory of their origin.

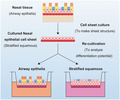

## 1 INTRODUCTION

Regenerative medicine using cultured cell sheets has the potential to be applied to a wide range of disorders that affect many different tissue types. For example, clinical studies have described the successful use of cultured cell sheets to treat patients with cardiac dysfunction^1^ or corneal damage.^2^ Regenerative medicine for otolaryngology is a rapidly developing field, and an important aim of our current research is to develop new treatments for severe middle ear diseases (i.e., cholesteatoma, hyperkeratosis is observed) using autologous cultured nasal epithelial cell sheets.^3^ The development of a new regenerative therapy requires an understanding of how a cell graft will behave after it has been transplanted into the target site. Native human epithelial tissue has wound healing properties because it can cover a wound surface and then increase in thickness.^4^, ^5^, ^6^ Therefore, we hypothesized that a cultured nasal epithelial cell sheet would have the ability to adhere to a wound, migrate and proliferate to cover the wound, and then differentiate into mucus cells and multiciliated cells that would promote mucociliary clearance. However, data are limited regarding the fate of cell sheets after transplantation because it is difficult to obtain suitable samples for analysis. Wang et al. recently reported the establishment of a two‐dimensional epithelial culture system that allowed them to study the behavior of colonic stem cells in vitro and gain insights into the process of re‐epithelization after epithelial injury.^7^ The authors suggested that in vitro analyses could help to improve our understanding of complex in vivo processes. We have developed an in vitro assay that involves grafting a cultured nasal epithelial cell sheet onto a collagen gel and then culturing the cell sheet for ≤1 week.^8^, ^9^ Using this assay, we have confirmed that the cells in a cultured nasal epithelial cell sheet have the potential to migrate and proliferate. However, we have not yet determined how the cell sheet would differentiate after reaching confluence.

The mucosal epithelial tissues of the nose and middle ear are lined by a pseudostratified columnar epithelium. The p63‐positive cells in the basal layer of this epithelium differentiate into various cell types that play important roles in mucociliary clearance: club cells that express secretoglobin family 1A member 1 (SCGB1A1), goblet cells that express mucin 5 AC (MUC5AC), and multiciliated cells that express both forkhead box J1 (FOXJ1) and acetyl‐α‐tubulin.^10^, ^11^, ^12^, ^13^ The differentiation processes that underlie the formation of this pseudostratified columnar epithelium are maintained by the surrounding environment,^14^ and exogenous factors can alter stem cell fate to generate a squamous epithelium.^15^, ^16^ However, no previous studies have investigated how the surrounding environment (i.e., the composition of the culture medium) affects the differentiation of cultured nasal epithelial cell sheets.

PneumaCult™‐Air‐Liquid Interface culture medium (P‐ALM) promotes the differentiation of human induced pluripotent stem (iPS) cells into pseudostratified columnar epithelium that contains functional multiciliated cells.^17^, ^18^ Keratinocyte Culture Medium (KCM), which is used during the fabrication of cultured nasal epithelial cell sheets,^8^, ^9^ induces cells derived from the skin epidermis to differentiate into keratinized stratified squamous epithelium containing cytokeratin‐1 (CK1)‐positive cells.^19^, ^20^ We hypothesized that these two culture media would drive the differentiation of cultured nasal epithelial cell sheets along different paths and thereby lead to differences in cell fate. Therefore, the aim of this study was to compare the differentiation of cultured nasal epithelial cell sheets cultured in P‐ALM or KCM under air‐liquid interface (ALI) conditions through analyses of surface morphology, protein expression and mRNA expression.

## 2 MATERIALS AND METHODS

### 2.1 Preparation and preservation of human epithelial tissue

This study was approved by the Institutional Review Board of The Jikei University, Tokyo, Japan (approval number: 26359). The study was conducted in accordance with the World Medical Association Declaration of Helsinki. The volunteers who donated nasal mucosal tissue were patients scheduled to undergo endoscopic sinus surgery at the Department of Otorhinolaryngology, The Jikei University School of Medicine. All volunteers provided informed consent for participation in this study and were confirmed not to be infected with human immunodeficiency virus, syphilis, hepatitis B virus or hepatitis C virus. A sample of nasal mucosal tissue was collected from the mucosa of the inferior nasal turbinate during endoscopic sinus surgery, and a sample of skin tissue was collected during tympanoplasty. The tissues were subjected to three different procedures depending on the experiment being performed: RNA extraction for quantitative polymerase chain reaction (qPCR), paraffin embedding for immunohistochemistry, and explant culture for the generation of cultured nasal epithelial cell sheets and expanded epidermal cells. Nasal mucosal tissues intended for RNA extraction were immersed in RNA later solution (Ambion, Austin, TX, USA) at 4°C for 1–2 days and then stored at −80°C until their use in the experiments.

### 2.2 Fabrication of cultured nasal epithelial cell sheets and preparation of epidermal cells and airway cells

Previously, we investigated to optimize explant culture and cell sheet culture of cultured nasal epithelial cell sheet.^8^, ^9^ In short, KCM comprised a 1:1 mixture of Dulbecco's modified Eagle medium (DMEM; Thermo Fisher Scientific, Waltham, MA, USA or Nacalai Tesque, Kyoto, Japan) and DMEM with Ham's F‐12 medium (Thermo Fisher Scientific) supplemented with 10% fetal bovine serum (Sigma‐Aldrich, St. Louis, MO, USA), 0.3 μM hydrocortisone (Saxizon®, Takeda Yakuhin Kogyo, Nihonbashi, Tokyo, Japan), 140.0 mU/ml insulin (Novo Nordisk, Bagsværd, Copenhagen, Denmark), 2.0 nM triiodothyronine (Sigma‐Aldrich), 0.2 μM epidermal growth factor (Higeta‐Shoyu, Nihonbashi, Tokyo, Japan), 1.0 nM cholera toxin (Wako Pure Chemical Industries, Dosyomachi, Osaka, Japan), 100 U/ml penicillin (Meiji Seika Pharma, Kyobashi, Tokyo, Japan), 69 μM streptomycin (Meiji Seika Pharma) and 0.4 μg/ml amphotericin B (Bristol‐Myers Squibb, New York City, NY, USA). A rho‐associated kinase (ROCK) inhibitor (10 μM Y‐27632, Wako Pure Chemical) was added to the KCM to promote cell expansion, and explant culture was performed as described in our previous article.^8^ Briefly, washed and disinfected nasal mucosal tissues were cultivated by explant culture for 13 days at 37°C. For sub‐culture, mouse fibroblasts 3T3‐J2 cells, which known of feeder cell of keratinocytes, were seeded at a density of 4.0 × 10^4^ cells/cm^2^ as a feeder layer, and epithelial cells were seeded at a density of 1.0–3.0 × 10^4^ cells/cm^2^ on the feeder cells for 4–7 days. During passaging, cells were collected after cultivation at 37°C and stored in cell freezing medium (Stem‐Cellbanker, GMP‐grade, Nippon Zenyaku Kogyo, Fukushima, Japan) at −80°C or in liquid nitrogen. Cell sheets were generated using temperature‐responsive cell culture substrate (CellSeed, Ōme, Tokyo, Japan) as described previously.^8^ Thawed cells were seeded at a density of 1.0 × 10^5^ cells/cm^2^ and cultured for 8 days at 37°C. The medium was changed to KCM without Y‐27632 on days 3, 5 and 7. The cells were detached as a cell sheet on day 8 by reducing the temperature to 20°C for 40 min.

### 2.3 ALI culture of cultured nasal epithelial cell sheets, skin epidermal cells and NHBE cells

Cultured nasal epithelial cell sheets were treated with 0.25% trypsin–EDTA, seeded on 12‐well cell culture inserts (Corning, Corning, NY, USA) at a density of 1.0 × 10^5^ cells/well, and cultured in KCM containing 1 μM Y‐27632 or P‐ALM (Veritas) containing 1 μM Y‐27632. In order to confirm the cell culture conditions were suitable for the induction of differentiation, we used human epidermal cells and human bronchial epithelial cells. In order to collect epidermal cells, surplus small pieces of skin tissues were collected at the time of wound closure in mastoidectomy (middle ear surgery). Same as nasal mucosal tissue, explant culture was performed in KCM containing 10 μM Y‐27632 for 13 days at 37°C, with medium changes performed on days 5 and 10. The epidermal cells derived from the skin were seeded (1.0 × 10^5^ cells/well) on 12‐well cell culture inserts and cultured in KCM supplemented with 1 μM Y‐27632. Moreover, we purchased three different batches of normal human bronchial epithelial cells (NHBE cells, Lonza, Basel, Switzerland) and expanded in PneumaCult™‐Ex medium (Veritas, Tokyo, Japan). The expanded NHBE cells were seeded (1.0 × 10^5^ cells) on 12‐well cell culture inserts and cultivated in P‐ALM containing 1 μM Y‐27632. For all cell types, submerged culture was performed for 1–2 weeks until the cells reached confluence followed by ALI culture for 2–3 weeks. The medium was changed every 2 or 3 days.

### 2.4 Immunohistology

Immunohistology was performed as described in our previous article.^21^ The tissues were fixed in 4% paraformaldehyde (Wako Pure Chemical or Nacalai Tesque), embedded in paraffin and sliced into 4‐μm sections. The sections were de‐paraffinized, and antigens were activated by autoclaving (121°C, 10 min) in citrate buffer (Histo‐VT One, Nacalai Tesque). Nonspecific reactions were prevented by blocking endogenous peroxidase with Peroxidase‐Blocking Solution (Dako, Carpinteria, CA, USA) and protein blocking with Blocking One Histo (Nacalai Tesque). The sections were incubated in phosphate‐buffered saline containing primary antibody (see Table S1) at 4°C overnight. Then, the sections were washed with phosphate‐buffered saline and incubated with horseradish peroxidase‐tagged secondary antibody (REAL EnVision™ Detection System, Dako) or goat anti‐rat IgG (HRP polymer) antibody (Abcam, Cambridge, UK) at room temperature for 1 h. Subsequently, the sections were treated with 3,3′‐diaminobenzidine (K5007; Dako). Nuclei were stained with hematoxylin.

### 2.5 qPCR

Frozen nasal tissue was thawed, cut into cubes (~1 mm^3^ in size) and homogenized in RLT Plus buffer, which is the lysis buffer provided in the RNeasy Plus Mini Kit (Qiagen, Hilden, Germany). Total RNA was extracted from nasal mucosal tissue and from cell sheets derived from nasal mucosal tissue using the RNeasy Plus Mini Kit. RNA was extracted from ALI cultured cells (re‐cultured cell sheets and bronchial epithelial cells) using the RNeasy Plus Micro Kit (Qiagen) in accordance with the manufacturer's protocol.

The cDNAs were prepared using a cDNA synthesis kit (SuperScript™ IV VILO™ Master Mix, Thermo Fisher Scientific). Comparisons between nasal tissue and cell sheets were made using cDNAs obtained from 10 ng of total RNA, and comparisons between cell sheets and ALI cultured cells were made using cDNAs obtained from 2.5 ng of total RNA. The cDNAs were mixed with DNA polymerase solution (Applied Biosystems, Foster City, CA, USA) and TaqMan probes (see Table S2). qPCR was performed using a StepOnePlus Real‐Time PCR system (Applied Biosystems). Since *RPLP0* and *RPLP1* were found to be suitable reference genes for comparisons of qPCR data between normal nasal tissue and nasal tissue with chronic rhinosinusitis,^22^ the present study used *RPLP0* for normalization of mRNA levels. Relative mRNA levels were quantified using the 2^−ΔΔCT^ method. For comparisons between nasal tissue and cell sheets, the mRNA levels were normalized to those of the nasal tissue; for comparisons between non‐re‐cultured cell sheets and re‐cultured cell sheets, the mRNA levels were normalized to those of the non‐re‐cultured cell sheets; for comparisons between cell sheets re‐cultured in P‐ALM and those re‐cultured in KCM, the mRNA levels were normalized to those of the cell sheets re‐cultured in P‐ALM.

### 2.6 Scanning electron microscopy (SEM)

SEM was performed as described by Manome et al.^23^ In short, cultured cells were fixed in 0.1 M phosphate buffer containing 1.2% glutaraldehyde. The samples were washed with 0.1 M phosphate buffer containing 5% sucrose after removal of the fixative solution. The specimens were fixed in 0.1 M phosphate buffer containing 1% osmium tetroxide and then dehydrated using ethanol and a critical point dryer (HCP‐2, Hitachi High‐Tech Co., Tokyo, Japan). The plates were coated with osmium (Os coater, HPC‐1SW, Vacuum Device Inc. Ibaragi, Japan). Images were captured using field‐emission SEM (Regulus8100, Hitachi, Tokyo, Japan).

### 2.7 Transmission electron microscopy (TEM)

TEM was carried out using a modification of a method reported previously.^8^ In short, cultured cells were fixed in 0.1 M phosphate buffer containing 2.0% glutaraldehyde and then treated with 0.1 M phosphate buffer containing 1% osmium tetroxide after removal of the fixative solution. The samples were dehydrated in ethanol, immersed in absolute propylene oxide and embedded in Epok 812 (Okensyoji, Tokyo, Japan). Trimming regions were confirmed using toluidine blue‐stained sections. The blocks were sliced into ~60‐nm sections with a diamond knife, mounted on grids, and stained with uranyl acetate and lead citrate. TEM images were captured at 80 kV (H‐7500, Hitachi, Tokyo, Japan).

### 2.8 Statistical analysis

Prism 9.0 software (GraphPad, Inc., La Jolla, CA, USA) was used for the analyses. Data are presented as the mean ± standard deviation (SD) and were compared between groups using the Mann–Whitney U test (two groups) or one‐way ANOVA with the Tukey post‐hoc test (more than two groups). A *p*‐value less than 0.05 was considered indicative of statistical significance.

## 3 RESULTS

### 3.1 Comparisons between nasal mucosal tissue and cell sheets derived from nasal mucosal tissue

**FIGURE 1:**
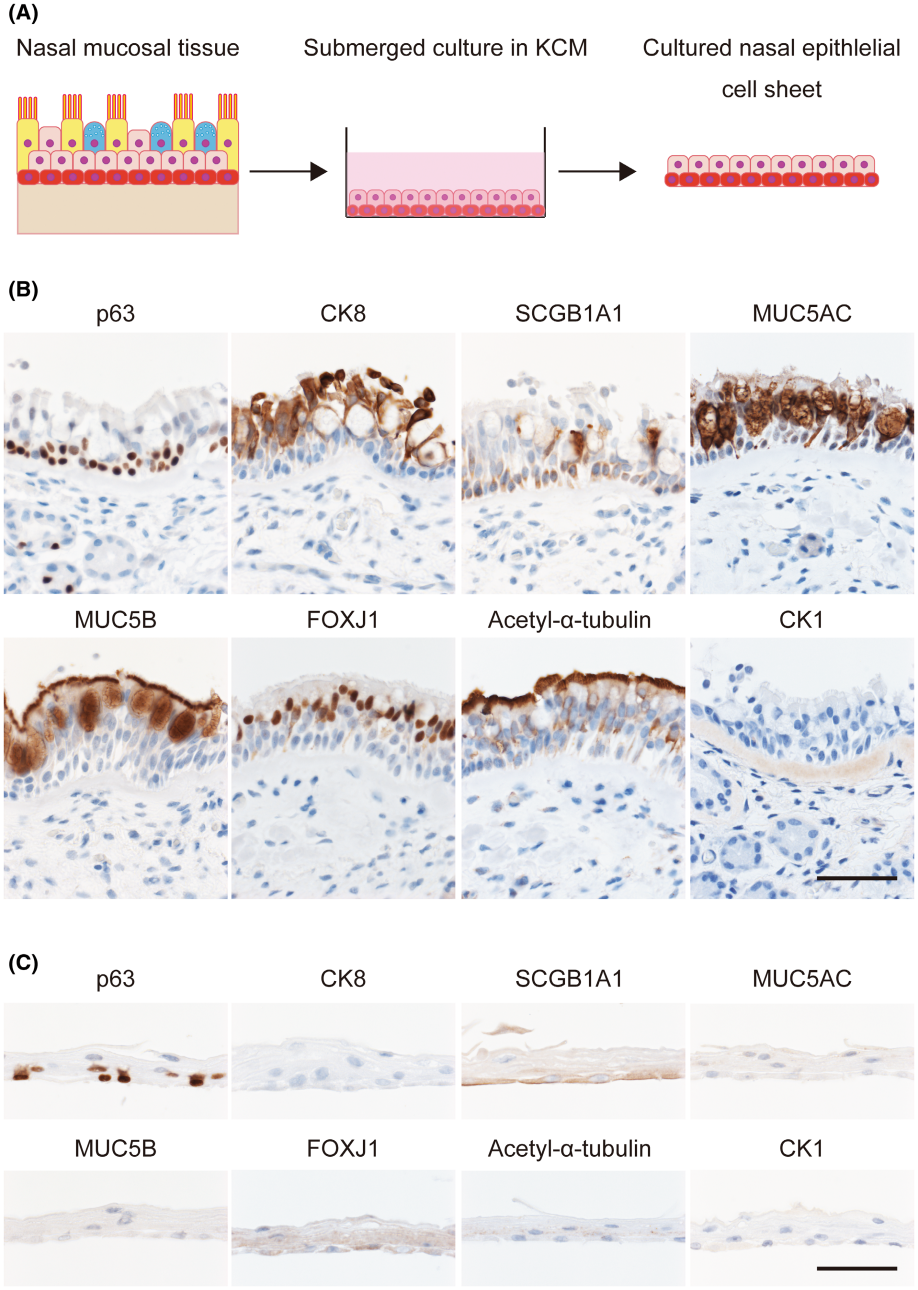
Comparisons between nasal mucosal tissue and cell sheets derived from nasal mucosal tissue. (A) Cultured nasal epithelial cell sheets were fabricated from human nasal mucosal tissue by culture in KCM. (B) Immunohistochemical analyses of nasal mucosal tissue. (C) Immunohistochemical analyses of cell sheets derived from nasal mucosal tissue. The top of each panel is labeled with the protein of interest (see also Table S1). Scale bar = 100 μm.

First, we examined the expressions of proteins related to airway epithelial cells. According to Atanasova and Reznikov, since mucins efflux from tissues or cultured cells, immunohistochemistry remains the gold standard method of analyzing protein expression in airway epithelial cells.^24^ Therefore, we performed immunohistochemical and qPCR analyses to compare the mRNA and protein expressions of several markers between nasal mucosal tissue and cell sheets derived from nasal mucosal tissue (Figures 1 and S1). The following markers were selected for analysis: p63 (encoded by the *TP63* gene), which is a candidate marker of basal epithelial stem cells^25^, ^26^; SCGB1A1 (encoded by *SCGB1A1*), which is a marker of club cells^27^; CK8 (encoded by *KRT8*), which is a marker of airway epithelial differentiation; CK1 (encoded by *KRT1*), which is a marker of keratinization; MUC5AC and MUC5B (encoded by *MUC5AC* and *MUC5B*), which are secreted mucins^24^; FOXJ1 (encoded by *FOXJ1*), which is a transcription factor in multiciliated cells; and acetyl‐α‐tubulin, which is a component of cilia.^15^, ^17^, ^28^

p63 was expressed in both nasal mucosal tissues and cultured nasal epithelial cell sheets, implying that cultured nasal epithelial cell sheets have the potential to differentiate (Figures 1 and S1). *TP63* expression was higher in cultured nasal epithelial cell sheets than in nasal mucosal tissues (*p* < 0.05; Figure S1), but this may have been due to differences in the cell populations (number of epithelial cell layers) rather than upregulation of the *TP63* gene. CK8 protein was expressed in nasal mucosal tissues but was barely detectable in cultured nasal epithelial cell sheets (Figure 1). By contrast, the mRNA expression of *KRT8* was comparable between nasal mucosal tissue and cultured nasal epithelial cell sheets (Figure S1), suggesting that the process of differentiation had begun in the cell sheets.

SCGB1A1 protein expression was observed in nasal mucosal tissue, indicating the presence of club cells, whereas only a small amount of SCGB1A1 expression was detected in the basal layer of the cell sheet (Figure 1). The mRNA expression of *SCGB1A1* was numerically much lower in cell sheets than in nasal mucosal tissue, although the difference was not statistically significant (Figure S1). MUC5AC and MUC5B were strongly expressed in the mucosal epithelium of nasal mucosal tissues but weakly expressed (if at all) in cultured nasal epithelial cell sheets (Figure 1). The mRNA expressions of these secreted mucins were significantly lower in cultured nasal epithelial cell sheets than in nasal mucosal tissue (*p* < 0.05; Figure S1), which agrees with the immunohistochemistry data. These results suggest that the cell sheets secrete little or no MUC5AC or MUC5B.

FOXJ1 and acetyl‐α‐tubulin protein levels appeared lower in cultured nasal epithelial cell sheets than in nasal mucosal tissue (Figure 1), and the *FOXJ1* mRNA expression level was significantly lower in cell sheets than in nasal mucosal tissue (*p* < 0.05; Figure S1). CK1 was not expressed in either type of nasal tissue. Therefore, the cultured nasal epithelial cell sheets were composed of proliferating basal cells and stratified squamous epithelial cells. These findings indicate that the characteristics of the cultured nasal epithelial cell sheets were closer to those of a stratified squamous epithelium than to those of a pseudostratified columnar epithelium.

### 3.2 Comparisons between non‐re‐cultured cultured nasal epithelial cell sheets and cell sheets re‐cultured in P‐ALM

Initially, we cultured commercially available airway epithelial cells (NHBE cells) in P‐ALM under an ALI in order to confirm that the cell culture conditions would promote the differentiation of airway epithelial cells and generate multiciliated cells. This culture method generated tissue with beating cilia, and immunohistochemistry confirmed its characteristics (Figure S2A). CK8 was expressed from the suprabasal layer to the uppermost layer. SCGB1A1, MUC5AC and MUC5B were expressed in the upper layer, and FOXJ1 and acetyl‐α‐tubulin were also observed in the upper layer. CK1 was not detected. The cilia on the upper surface were clearly observed by SEM (Figure S2B). TEM demonstrated that the cilia contained nine doublet microtubules with dynein arms surrounding two central microtubules (Figure S2C), which is the same structure as that found in the cilia of normal airway cells.^29^ Images of airway‐derived cells captured with a high‐speed camera demonstrated ciliary beating (Movie 1). The above results indicate that this culture method promotes the differentiation of airway cells and the generation of multiciliated cells.

**FIGURE 2:**
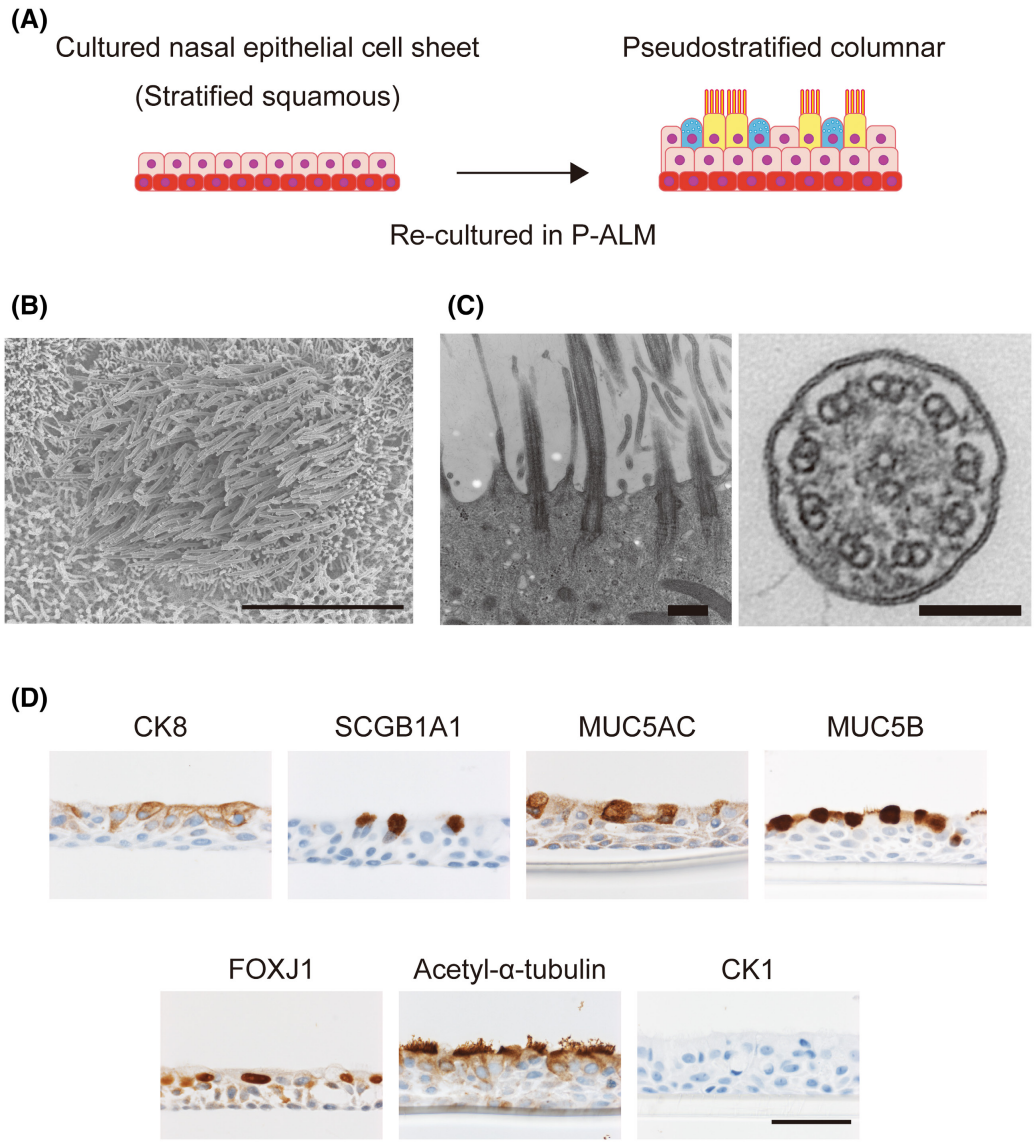
Characterization of cultured nasal epithelial cell sheets re‐cultured in P‐ALM under an ALI. (A) Cultured nasal epithelial cell sheets were trypsinized and then re‐cultured on 12‐well cell culture inserts in P‐ALM. (B) Representative SEM image. Scale bar = 10 μm. (C) Representative TEM images of a cultured nasal epithelial cell sheet re‐cultured in P‐ALM under an ALI. Scale bar = 500 nm (left) and 100 nm (right). (D) Immunohistochemical analyses of cell sheets re‐cultured in P‐ALM under an ALI. The top of each panel is labeled with the protein of interest. Scale bar = 50 μm.

Next, nasal cell sheets were re‐cultured in P‐ALM under an ALI, and their phenotype was analyzed (Figure 2A). Cilia were clearly observed by SEM (Figure 2B), and TEM confirmed the presence of nine doublet microtubules surrounding two central microtubules (Figure 2C). Ciliary beating was also observed (Movie 2). Immunohistochemistry revealed that CK8 (an epithelial differentiation marker), SCGB1A1 (found in club cells), MUC5AC and MUC5B (secreted mucins) were all expressed in the re‐cultured cell sheet (Figure 2D). The expression of FOXJ1 (a marker of multiciliated cells) was noticeably higher after re‐culture of the cell sheet than before re‐culture, and there was little or no expression of CK1 (Figure 2D).

### 3.3 Comparisons between non‐re‐cultured cultured nasal epithelial cell sheets and cell sheets re‐cultured in KCM

Skin epithelial cells were cultured in KCM to confirm that the cell culture conditions were suitable for the induction of keratinization. Proteins related to airway epithelium (CK8, SCGB1A1, MUC5AC, MUC5B, FOXJ1, and acetyl‐α‐tubulin) were not detected (Figure S3). Immunohistochemistry analyses confirmed the presence of peeling/shedding at the tissue surface as well as the expression of CK1 (a marker of terminal differentiation in keratinizing epithelia) in the upper layer. These observations provide evidence that culture in KCM induces epidermal cells to undergo keratinization.

**FIGURE 3:**
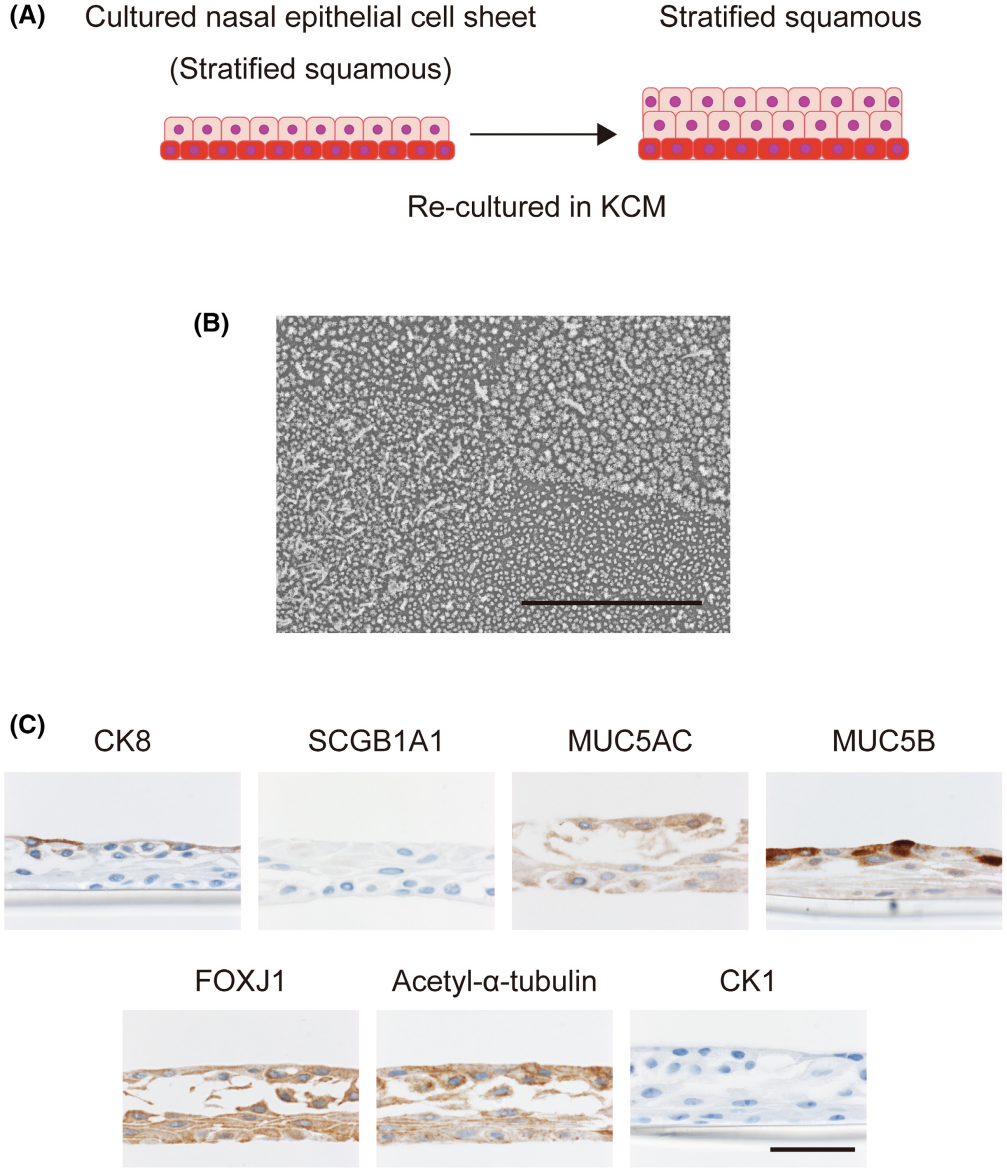
Characterization of cultured nasal epithelial cell sheets re‐cultured in KCM. (A) Cultured nasal epithelial cell sheets were trypsinized and then re‐cultured on 12‐well cell culture inserts in KCM. (B) Representative SEM image. Scale bar = 10 μm. (C) Immunohistochemical analyses of cell sheets re‐cultured in KCM. The top of each panel is labeled with the protein of interest. Scale bar = 50 μm.

In the subsequent experiments, cultured nasal epithelial cell sheets were re‐cultured in KCM with use of an ALI once the cells had reached confluence (Figure 3A). SEM demonstrated that most of the scanned region was occupied by squamous cells, and multiciliated cells were observed in only one of six cases (Figure 3B). Immunohistochemical analyses revealed that CK8 was weakly expressed and SCGB1A1 was not expressed in the upper surface of the re‐cultured cell sheet (Figure 3C). MUC5AC and MUC5B were expressed in some cells of the re‐cultured cell sheet, although these cells did not have shapes that were characteristic of goblet cells. FOXJ1 (a ciliary marker) was rarely detected in cell sheets re‐cultured in KCM (one in six cases). CK1 was not detected in the re‐cultured cell sheet, indicating that differentiation to keratinized cells had not occurred (Figure 3C). Therefore, cells in a cultured nasal epithelial cell sheet are able to differentiate into mucus‐producing cells and squamous epithelial cells when cultured in KCM, but differentiation into keratinized cells and multiciliated cells occurs only rarely under these conditions.

### 3.4 Comparisons between cell sheets re‐cultured in P‐ALM and cell sheets re‐cultured in KCM

**FIGURE 4:**
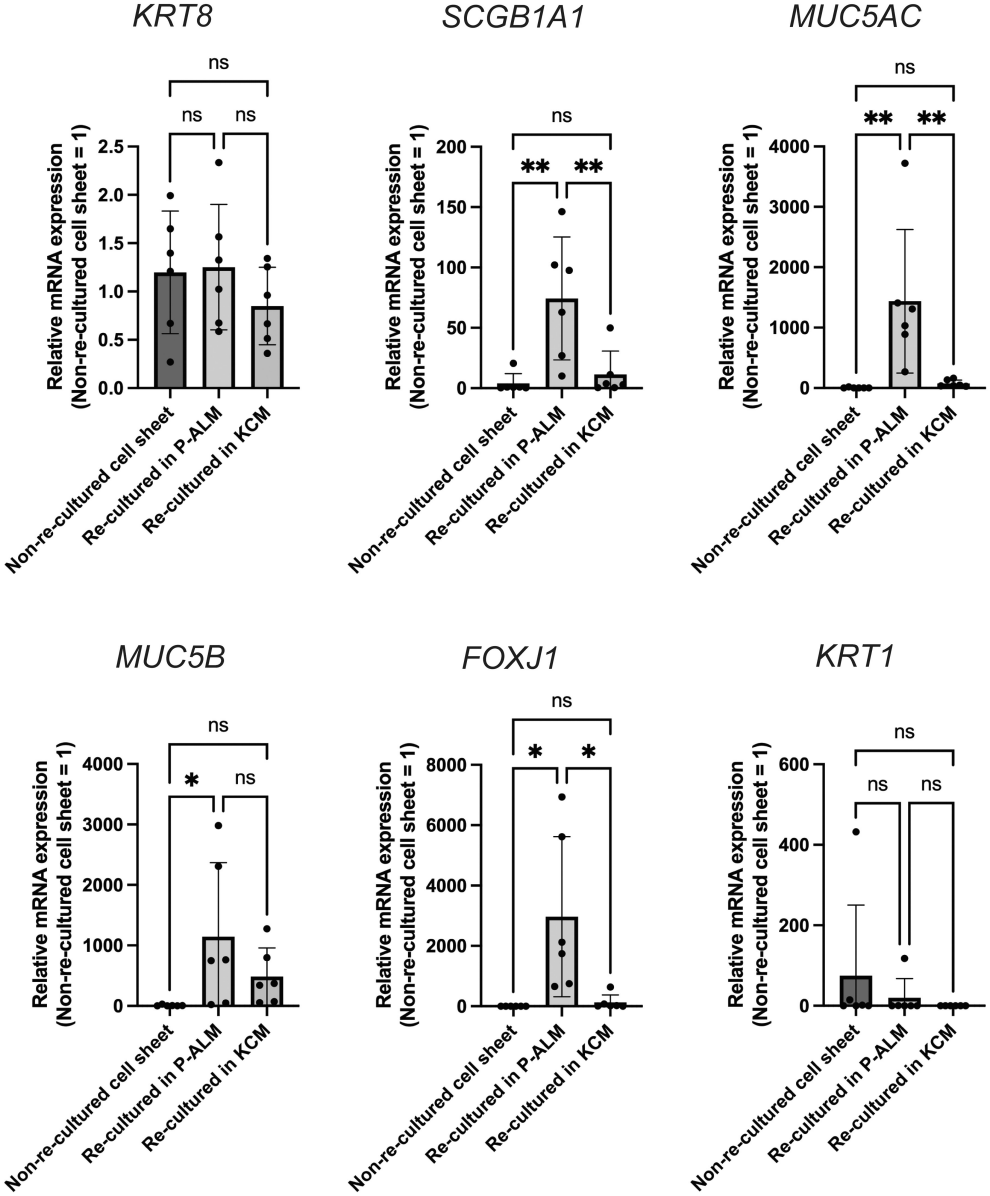
Comparisons of the mRNA expressions of various genes between non‐re‐cultured cultured nasal epithelial cell sheets, cell sheets re‐cultured in P‐ALM and cell sheets re‐cultured in KCM. The mRNA expressions were measured using qPCR. The top of each panel is labeled with the gene of interest. Values are expressed as the mean ± SD (*n* = 6). *p < 0.05, **p < 0.01.

The mRNA expressions of *KRT8* and *KRT1* were not significantly different between cultured nasal epithelial cell sheets re‐cultured in P‐ALM and those re‐cultured in KCM (Figure 4). On the other hand, the mRNA expressions of *SCGB1A1*, *MUC5AC*, and *FOXJ1* were significantly higher; MUC5B was tended to be higher in cultured nasal epithelial cell sheets re‐cultured in P‐ALM than in cell sheets re‐cultured in KCM (*p* < 0.01, *p* < 0.01, *p* < 0.05, ns, respectively), which supports the findings of the immunohistochemical analyses. These observations demonstrate that the cell culture conditions have a major effect on the phenotype of a cultured nasal epithelial cell sheet. The reproducibility of the findings for FOXJ1 expression was confirmed in a series of four independent immunohistochemistry experiments (Figure S4).

## 4 DISCUSSION

The present study investigated the differentiation potential of human cultured nasal epithelial cell sheets cultured in two different media. The findings show that the culture conditions have a profound impact on cultured nasal epithelial cell sheet phenotype. Furthermore, the cells in cultured nasal epithelial cell sheets have the potential to differentiate into club cells, mucus cells, and multiciliated cells (all of which are found in the pseudostratified columnar epithelium of the airways) if appropriate culture conditions are utilized.

The procedure used in this study to generate cell sheets from nasal mucosal tissue involved submerged culture in KCM containing a ROCK inhibitor and the use of feeder cells.^9^ Submerged culture induces little differentiation of airway cells to goblet cells and multiciliated cells, in contrast to ALI culture.^30^ KCM contains epithelial growth factor, which is a known regulator of stratified squamous epithelial cells but a downregulator of multiciliated cells.^15^ ROCK inhibitors and feeder cells both help to maintain the undifferentiated state of stem cells.^31^, ^32^, ^33^ Thus, submerged culture in KCM may affect the characteristics of cultured nasal epithelial cell sheets that contain many squamous epithelial cells and p63‐positive cells (Figure 1).

**FIGURE 5:**
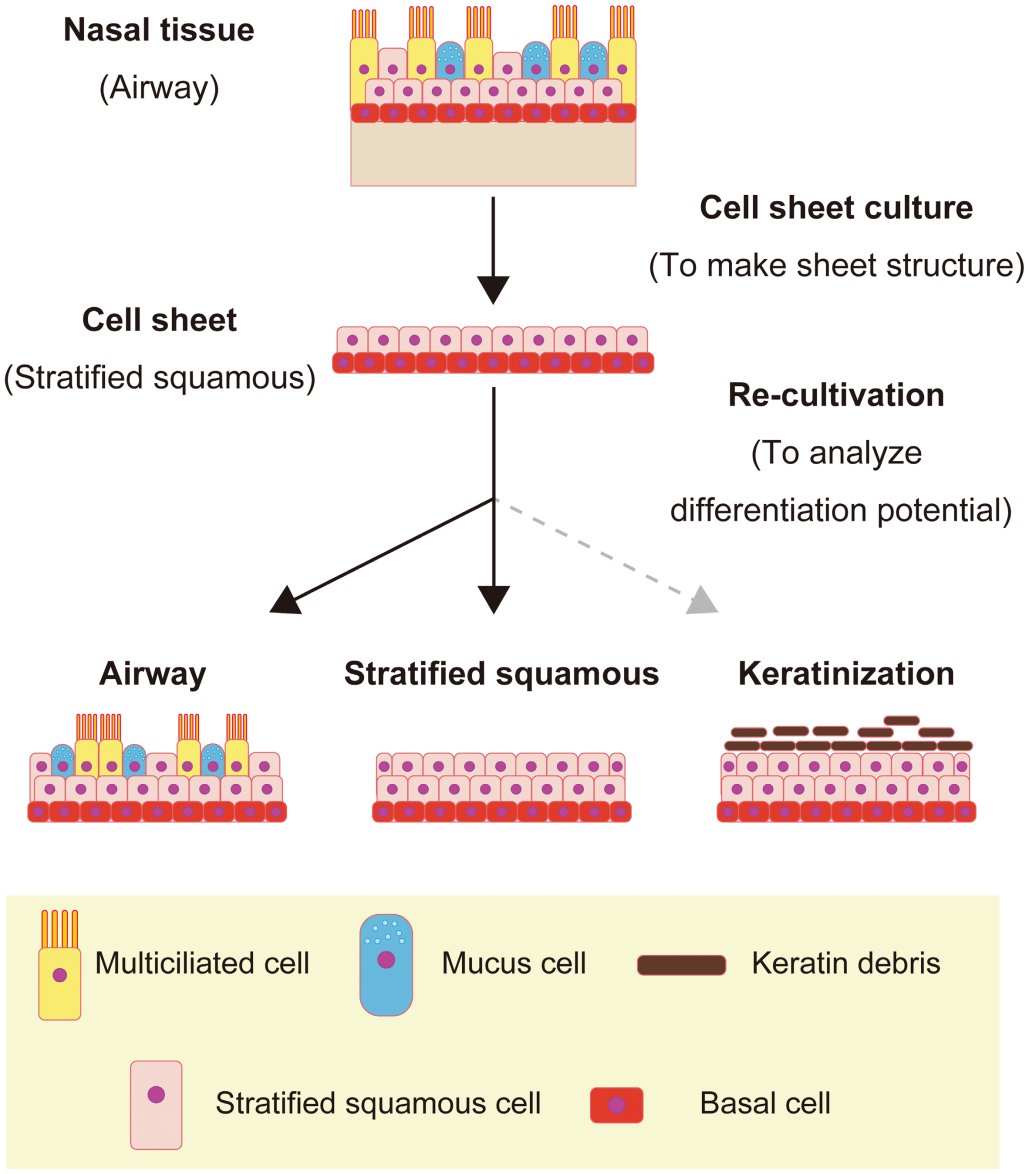
Schematic diagram summarizing the differentiation potential of cultured nasal epithelial cell sheets

Claudinot et al. demonstrated that p63‐positive cells derived from various epithelial cell types can contribute to hair follicles, sebaceous glands and epidermis renewal when exposed to a skin microenvironment.^26^ Cells derived from the skin, oral mucosa, esophagus and bladder formed normal skin‐like structures that included squamous epithelium, hair follicles and hair. By contrast, p63‐positive cells derived from the trachea (a major airway) and ureter were unable to form hair follicles and hair, although they could be differentiated into squamous epithelium. The results of the study by Claudinot et al. suggest that although the fate of epithelial stem cells is substantially affected by exogenous factors, “memory” of their origin also exerts an influence as an endogenous factor. Therefore, we re‐cultured cultured nasal epithelial cell sheets in two different media in order to investigate whether exogenous factors affected cellular differentiation. Although cultured nasal epithelial cell sheets before re‐cultivation formed a squamous epithelium with basal cells, re‐culture in P‐ALM under an ALI resulted in the cell sheets expressing SCGB1A1, MUC5AC, FOXJ1, and acetyl‐α‐tubulin (Figure 2). These observations demonstrate that cultured nasal epithelial cell sheets have the potential to differentiate into club cells, goblet cells and functional multiciliated cells when cultured in P‐ALM under ALI conditions. On the other hand, cells in cultured nasal epithelial cell sheets did not differentiate into CK1‐positive cells after re‐cultivation in KCM (Figure 3) even though skin‐derived epithelial cells were able to differentiate into CK1‐positive cells under these conditions (Figure S3). Therefore, our results support the proposal that stem cell “memory” allows cultured nasal epithelial cell sheets to differentiate into a pseudostratified columnar epithelium or a mucosal stratified squamous epithelium but not a keratinized epithelium (Figure 5).

Understanding the functional behavior of transplanted cells after grafting onto a wound requires that the cells are analyzed after wound repair has occurred. Counter et al. reported that cultured epithelial autografts could be used to regenerate skin on patients with burns, although the autografts had shorter telomeres than non‐cultured skin at 10 or more years after transplantation, likely due to repeated cell division during culture of the autograft.^34^ Another study showed that oral mucosal cell sheets were able to regenerate the transparent stratified squamous epithelium of the cornea in patients with corneal defects.^2^ Furthermore, cultured nasal epithelial cell sheets were demonstrated to exert a regenerative effect on the middle ear mucosa and maintain middle ear aeration in patients with middle ear cholesteatoma (hyperkeratosis is observed) or adhesive otitis media, implying that the wound created during surgery had become covered by epithelial tissue.^3^, ^35^ Preclinical studies investigating and optimizing the differentiation potential of cultured nasal epithelial cell sheets would likely help to improve the efficacy of these grafts in the clinical setting. Multiciliated cells are located in the tympanic cavity, especially around the eustachian tube, and are thought to play an important role in the removal of foreign substances from the nasal cavity.^36^ Chronic inflammation of the basal cells of airway epithelium reduces their ability to differentiate into multiciliated cells even after the inflammatory stimuli have been removed.^37^, ^38^ Since we have demonstrated that cultured nasal epithelial cell sheets have the potential to differentiate into goblet cells and multiciliated cells, we predict that transplanted cultured nasal epithelial cell sheets would help to regenerate mucociliary clearance in the middle ear. Mucociliary clearance is also important in the paranasal sinuses and trachea, raising the possibility that cultured nasal epithelial cell sheets could be used to treat chronic sinusitis and asthma.

This study has two main limitations. First, we did not investigate the long‐term viability or therapeutic potential of cultured nasal epithelial cell sheets in animal experiments. A recent study found that multiciliated cells derived from iPS cells survive around the eustachian tube for at least 2 weeks.^18^ Polymer and nanoparticles that include vascular endothelial growth factor enabled cardiomyocyte sheet to extend survival of cells in vivo.^39^ ROCK inhibitor enhanced therapeutic effect of cultured cells on corneal endothelial regeneration.^40^, ^41^ Therefore, we speculate that in addition to cell sheet, polymers, growth factors, and other reagents as micro‐environment would be important to exert mucociliary clearance effect by multi‐ciliated cells, which differentiated from cells of the cultured nasal epithelial cell sheet.

Second, we did not analyze the medium conditioned by the re‐cultured cell sheets to explore whether cultured nasal epithelial cell sheets might exert paracrine effects. Cardiomyocyte cell sheets derived from human iPS cells are able to integrate into a beating heart, but the main beneficial action is considered to be vascularization of the surrounding region through a paracrine effect.^42^, ^43^ In addition, extracellular vesicles (exosomes) are thought to have important roles in wound healing.^44^, ^45^ Hence, we would expect cultured nasal epithelial cell sheets to exert paracrine effects after grafting. In vitro analyses of the culture medium might help to enhance our understanding of the paracrine effects of cell sheets. Additional studies are needed to explore these issues.

## 5 CONCLUSION

In conclusion, the conditions used for cultivation exert a major effect on the phenotype of a re‐cultured cultured nasal epithelial cell sheet (Figure 5). Notably, the cells in a cultured nasal epithelial cell sheet have the potential to differentiate into club cells, mucus cells and multiciliated cells when cultured in P‐ALM under an ALI. We anticipate that our findings and methodological approach will facilitate clarification of the fate of a transplanted cell graft intended for use in regenerative medicine.

## AUTHOR CONTRIBUTIONS

Yoshiyuki Kasai designed and carried out the experiments, analyzed the data, and drafted the manuscript. Tsunetaro Morino designed the methods used for cell sheet culture and explant culture and performed data analysis. Tsuguhisa Nakayama carried out the cell culture experiments and drafted the manuscript. Kazuhisa Yamamoto and Hiromi Kojima revised the manuscript and supervised this project.

## CONFLICT OF INTEREST

The authors declare no potential conflicts of interest with respect to the research, authorship, and/or publication of this article.

## Supporting information


Table S1.

Table S2.

Supplementary S1.

Figure S1.

Figure S2.

Figure S3.

Figure S4.

Movie 1.

Movie 2.

## Data Availability

The data that support the findings of this study are available from the corresponding author, Y.K., upon reasonable request.
