# Supplementary material for: Analysis of the potential of human cultured nasal epithelial cell sheets to differentiate into airway epithelium
Source: FASEB Bioadv. 2022 Dec 19;5(3):89–100. doi: 10.1096/fba.2022-00106 (PMC9983074; doi:10.1096/fba.2022-00106)
Supplement: Supplementary file 1 — Table S1. [file FBA2-5-89-s007.docx]

| **Table S1. Antibodies used in the immunohistological analyses.** | | | | |
| --- | --- | --- | --- | --- |
| Antibody | Cat. no. | Source | Dilution | Activation |
| Mouse anti-acetyl-α-tubulin monoclonal antibody (acetyl K40) [6-11B-1] | ab24610 | Abcam, Cambridge, UK | 1:32000 | pH 7, heat |
| Mouse anti-FOXJ1 monoclonal antibody [2A5] | 14-9965-82 | Thermo Fisher Scientific, Waltham, MA, USA | 1:500 | pH 7, heat |
| Mouse anti-cytokeratin 1 (CK1) monoclonal antibody [34βB4] | ENZ-30904 | Enzo Life Sciences, Farmingdale, NY, USA | 1:100 | ProK |
| Rabbit anti-cytokeratin 4 (CK4) monoclonal antibody [6B10] | ab9004 | Santa Cruz Biotechnology, Dallas, TX, USA | 1:20 | pH 9, heat |
| Mouse anti-cytokeratin 5 (CK5) monoclonal antibody [XM26] | ab17130 | Abcam, Cambridge, UK | 1:5000 | pH 9, heat |
| Mouse anti-cytokeratin 8 (CK8) monoclonal antibody [M20] | sc-52324 | Santa Cruz Biotechnology, Dallas, TX, USA | 1:100 | pH 7, heat |
| Mouse anti-cytokeratin 14 (CK14) monoclonal antibody [LL001] | sc-53253 | Santa Cruz Biotechnology, Dallas, TX, USA | 1:20 | pH 7, heat |
| Rabbit anti-cytokeratin 17 (CK17) monoclonal antibody [EP1623] | ab109725 | Abcam, Cambridge, UK | 1:100 | pH 7, heat |
| Mouse anti-p63 monoclonal antibody [4A4] | ab735 | Abcam, Cambridge, UK | 1:100 | pH 7, heat |
| Rat anti-uteroglobin/SCGB1A1 monoclonal antibody | MAB4218-SP | R&D Systems, Minneapolis, MN, USA | 3:100 | pH 7, heat |
| Mouse anti-MUC1 monoclonal antibody [VU4H5] | sc-7313 | Santa Cruz Biotechnology, Dallas, TX, USA | 1:200 | pH 7, heat |
| Mouse anti-MUC16 monoclonal antibody [X325] | sc-52096 | Santa Cruz Biotechnology, Dallas, TX, USA | 1:100 | pH 7, heat |
| Mouse anti-MUC5AC monoclonal antibody [CLH2] | ab77995 | Abcam, Cambridge, UK | 1:1200 | pH 7, heat |
| Mouse anti-MUC5B monoclonal antibody | NBP1-92151 | Novus Biologicals LLC, Centennial, CO, USA | 1:1000 | pH 7, heat |
