# Supplementary material for: Analysis of the potential of human cultured nasal epithelial cell sheets to differentiate into airway epithelium
Source: FASEB Bioadv. 2022 Dec 19;5(3):89–100. doi: 10.1096/fba.2022-00106 (PMC9983074; doi:10.1096/fba.2022-00106)
Supplement: Supplementary file 2 — Table S2. [file FBA2-5-89-s001.docx]

| **Table S2. TaqMan probes used in the qPCR analyses.** | | | |
| --- | --- | --- | --- |
| Gene | Assay ID | Cat. no. | Dye |
| *KRT1* | Hs00196158_m1 | 4453320 | FAM-MGB |
| *KRT4* | Hs00361611_m1 | 4453320 | FAM-MGB |
| *KRT5* | Hs00361185_m1 | 4453320 | FAM-MGB |
| *KRT8* | Hs01595539_g1 | 4448892 | FAM-MGB |
| *KRT14* | Hs00265033_m1 | 4453320 | FAM-MGB |
| *KRT17* | Hs00356958_m1 | 4448892 | FAM-MGB |
| *PRLP0* | Hs00420895_gH | 4331182 | FAM-MGB |
| *FOXJ1* | Hs00230964_m1 | 4453320 | FAM-MGB |
| *Tp63* | Hs00978340_m1 | 4331182 | FAM-MGB |
| *MUC1* | Hs00159357_m1 | 4453320 | FAM-MGB |
| *MUC5AC* | Hs01365616_m1 | 4331182 | FAM-MGB |
| *MUC5B* | Hs00861595_m1 | 4331182 | FAM-MGB |
| *MUC16* | Hs01065175_m1 | 4448892 | FAM-MGB |
| *SCGB1A1* | Hs00171092_m1 | 4453320 | FAM-MGB |
