# Supplementary material for: Analysis of the potential of human cultured nasal epithelial cell sheets to differentiate into airway epithelium
Source: FASEB Bioadv. 2022 Dec 19;5(3):89–100. doi: 10.1096/fba.2022-00106 (PMC9983074; doi:10.1096/fba.2022-00106)
Supplement: Supplementary file 3 — Supplementary S1. [file FBA2-5-89-s005.docx]

**Supporting Information**

**Analysis of the potential of human nasal mucosal cell sheets to differentiate into airway epithelium**

**Yoshiyuki Kasai^1*^, Tsunetaro Morino^1^, Tsuguhisa Nakayama^1,2^, Kazuhisa Yamamoto^1^, Hiromi Kojima^1*^**

Affiliations: ^1^ Department of Otorhinolaryngology, The Jikei University School of Medicine, Tokyo, Japan, ^2^ Department of Otorhinolaryngology, Head and Neck Surgery, Dokkyo Medical University, Tochigi, Japan

* Corresponding authors: Yoshiyuki Kasai Ph.D. and Hiromi Kojima M.D. Ph.D.

Department of Otorhinolaryngology, Jikei University School of Medicine, 3-25-8 Nishi-Shinbashi, Minato-ku, Tokyo 105-8461, Japan

Institutional email address: [ykasai@jikei.ac.jp](mailto:ykasai@jikei.ac.jp), [kojimah@jikei.ac.jp](mailto:kojimah@jikei.ac.jp)

Tel: +81-3-3433-1111, ext. 3601

Fax: +81-3-3578-9208

**Figure S1.** **Comparisons of the mRNA expressions of various genes between nasal mucosal tissue and cultured nasal epithelial cell sheets.** The mRNA expressions were measured using qPCR (TaqMan probes are shown in Table S1). The top of each panel is labeled with the gene of interest. Values are expressed as the mean ± SD (*n* = 6). **P* < 0.05, ***P* < 0.01.

**Figure S2.** **Characterization of NHBE airway cells cultured in P-ALM.** (A) Immunohistochemical analyses of NHBE airway cells cultured in P-ALM under an ALI. The top of each panel is labeled with the gene of interest. Scale bar = 50 μm. (B) Representative SEM image of an airway cell cultured in P-ALM under an ALI. Scale bar = 10 μm. (C) Representative TEM images of an airway cell cultured in P-ALM under an ALI. Scale bars = 500 nm (left) and 100 nm (right).

**Figure S3. Characterization of epidermal cells cultured in KCM.** Immunohistochemical analyses of epidermal cells cultured in KCM. The top of each panel is labeled with the gene of interest. Scale bar = 50 μm.

**Figure S4.** **Reproducibility of FOXJ1 expression in a series of experiments.** Case 1 is also shown in Figures 1−3. Scale bar = 50 μm.

**Movie 1.** Video images captured by a high-speed camera showing ciliary beating in cultured NHBE airway cells (related to Fig. S2A).

**Movie 2.** Video images captured by a high-speed camera showing ciliary beating in cultured nasal epithelial cell sheets cultured in P-ALM under ALI (related to Fig. 2).
