# Supplementary material for: Analysis of the potential of human cultured nasal epithelial cell sheets to differentiate into airway epithelium
Source: FASEB Bioadv. 2022 Dec 19;5(3):89–100. doi: 10.1096/fba.2022-00106 (PMC9983074; doi:10.1096/fba.2022-00106)
Supplement: Supplementary file 4 — Figure S1. [file FBA2-5-89-s004.pdf]

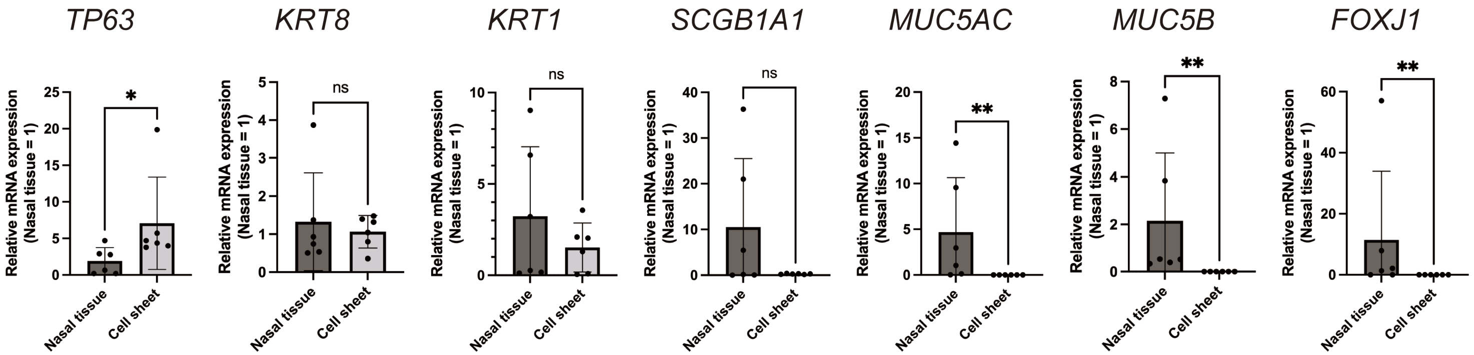

Figure S1. Comparisons of the mRNA expressions of various genes between nasal mucosal tissue and cultured nasal epithelial cell sheets.

The mRNA expressions were measured using qPCR (TaqMan probes are shown in Table S1).

The top of each panel is labeled with the gene of interest. Values are expressed as the mean ± SD (n = 6).

*P < 0.05, **P < 0.01.
