# Supplementary material for: Analysis of the potential of human cultured nasal epithelial cell sheets to differentiate into airway epithelium
Source: FASEB Bioadv. 2022 Dec 19;5(3):89–100. doi: 10.1096/fba.2022-00106 (PMC9983074; doi:10.1096/fba.2022-00106)
Supplement: Supplementary file 5 — Figure S2. [file FBA2-5-89-s008.pdf]

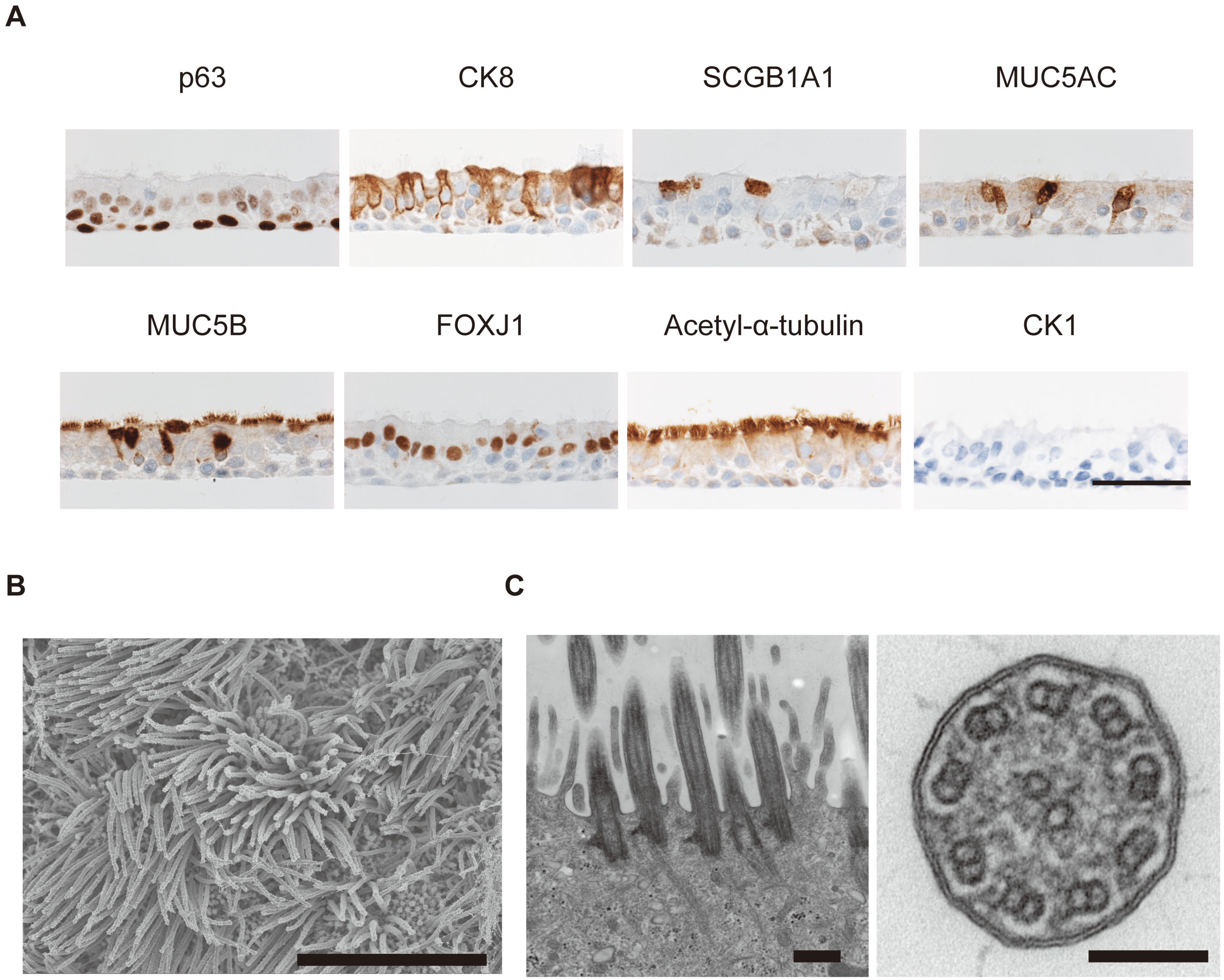

Figure S2. Characterization of NHBE airway cells cultured in P-ALM. (A) Immunohistochemical analyses of NHBE airway cells cultured in P-ALM under an ALI. The top of each panel is labeled with the gene of interest. Scale bar = 50 μm. (B) Representative SEM image of an airway cell cultured in P-ALM under an ALI. Scale bar = 10 μm. (C) Representative TEM images of an airway cell cultured in P-ALM under an ALI. Scale bars = 500 nm (left) and 100 nm (right).
