# Supplementary material for: Analysis of the potential of human cultured nasal epithelial cell sheets to differentiate into airway epithelium
Source: FASEB Bioadv. 2022 Dec 19;5(3):89–100. doi: 10.1096/fba.2022-00106 (PMC9983074; doi:10.1096/fba.2022-00106)
Supplement: Supplementary file 6 — Figure S3. [file FBA2-5-89-s002.pdf]

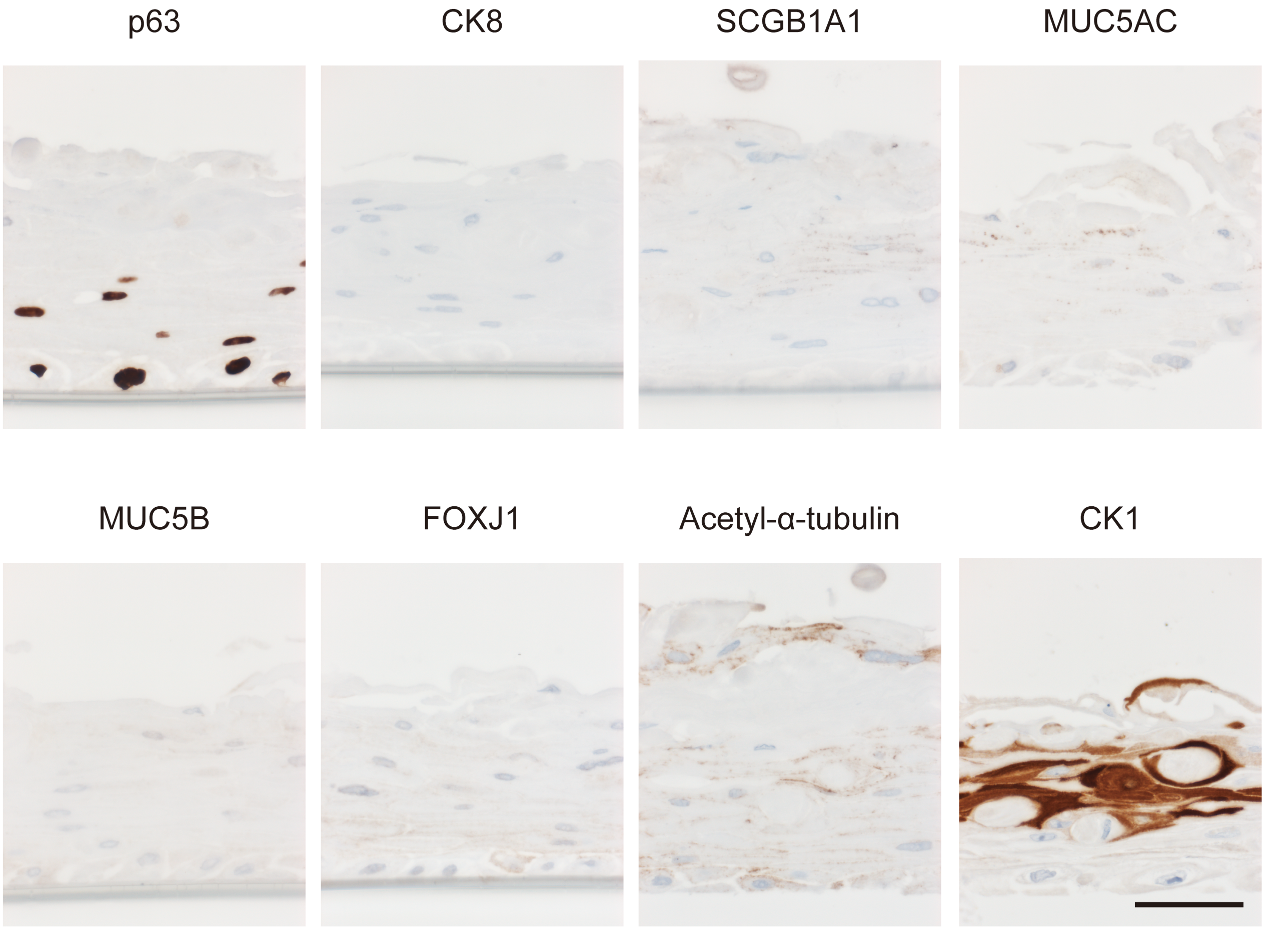

Figure S3. Characterization of epidermal cells cultured in KCM. Immunohistochemical analyses of epidermal cells cultured in KCM. The top of each panel is labeled with the gene of interest. Scale bar = 50 μm.
