# Supplementary material for: Analysis of the potential of human cultured nasal epithelial cell sheets to differentiate into airway epithelium
Source: FASEB Bioadv. 2022 Dec 19;5(3):89–100. doi: 10.1096/fba.2022-00106 (PMC9983074; doi:10.1096/fba.2022-00106)
Supplement: Supplementary file 7 — Figure S4. [file FBA2-5-89-s003.pdf]

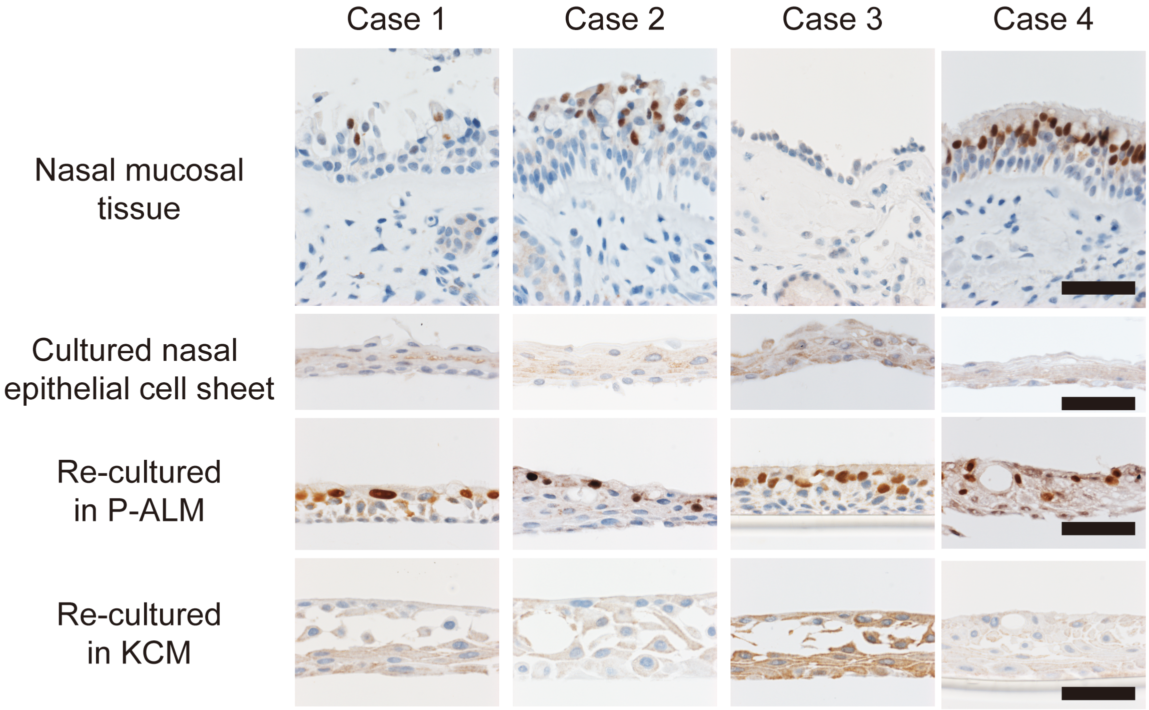

Figure S4. Reproducibility of FOXJ1 expression in a series of experiments. Case 1 is also shown in Figures 1–3. Scale bar = 50 μm.
